# In Vitro Synergistic Activity of Antimicrobial Combinations against Carbapenem- and Colistin-Resistant *Acinetobacter baumannii* and *Klebsiella pneumoniae*

**DOI:** 10.3390/antibiotics12010093

**Published:** 2023-01-05

**Authors:** Paraskevi Mantzana, Efthymia Protonotariou, Angeliki Kassomenaki, Georgios Meletis, Areti Tychala, Eirini Keskilidou, Maria Arhonti, Charikleia Katsanou, Aikaterini Daviti, Olga Vasilaki, Georgia Kagkalou, Lemonia Skoura

**Affiliations:** Department of Microbiology, School of Medicine, AHEPA University Hospital, Aristotle University of Thessaloniki, S. Kiriakidi str. 1, 54636 Thessaloniki, Greece

**Keywords:** synergistic activity, colistin, meropenem, imipenem, ceftazidime/avibactam, rifampicin, daptomycin, fosfomycin, aztreonam, amikacin

## Abstract

Polymyxins are commonly used as the last resort for the treatment of MDR *Acinetobacter baumannii* and *Klebsiella pneumoniae* nosocomial infections; however, apart from the already known toxicity issues, resistance to these agents is emerging. In the present study, we assessed the in vitro synergistic activity of antimicrobial combinations against carbapenem-resistant and colistin-resistant *A. baumannii* and *K. pneumoniae* in an effort to provide more options for their treatment. Two hundred *A. baumannii* and one hundred and six *K. pneumoniae* single clinical isolates with resistance to carbapenems and colistin, recovered between 1 January 2021 and 31 July 2022,were included. *A. baumannii* were tested by the MIC test strip fixed-ratio method for combinations of colistin with either meropenem or rifampicin or daptomycin. *K. pneumoniae* were tested for the combinations of colistin with meropenem and ceftazidime/avibactam with aztreonam. Synergy was observed at: 98.99% for colistin and meropenem against *A. baumannii*; 91.52% for colistin and rifampicin; and 100% for colistin and daptomycin. Synergy was also observed at: 73.56% for colistin and meropenem against *K. pneumoniae* and; and 93% for ceftazidime/avibactam with aztreonam. The tested antimicrobial combinations presented high synergy rates, rendering them valuable options against *A. baumannii* and *K. pneumoniae* infections.

## 1. Introduction

Infections caused by antimicrobial-resistant Gram-negative pathogens are a healthcare issue of major importance and are associated with poor patient outcomes [1,2]. *Acinetobacter baumannii* and *Klebsiella pneumoniae* often develop mechanisms to evade the action of antimicrobials and can acquire genes encoding for antimicrobial resistance mechanisms. Among them, carbapenemases are the most clinically important [3]. The extent of resistance of each isolate may vary; therefore, different definitions may be applied: multi-drug resistant (MDR) refers to an isolate that is resistant to three or more antimicrobial categories, extensively drug resistant (XDR) refers to an isolate that is susceptible to only one last resort antimicrobial and pan-drug resistant (PDR) refers to an isolate that is resistant to all available antimicrobials [4].

The presence and spread of MDR, XDR and even PDR Gram-negatives is dramatically limiting the treatment options for infections caused by these pathogens, whereas the pipeline of new antimicrobials is slow and novel compounds including tigecycline, eravacycline and cefiderocol do not always meet the expectations [5,6,7]. Current β-lactams combined with novel β-lactamase inhibitors provide some solutions especially against non-metallo-β-lactamase producers [8], but they are not applicable in all cases, and resistance has already emerged [9,10]. Monotherapy with formerly abandoned antimicrobials such as fosfomycin and polymyxins is another option. However, it presents limitations including dosing issues for fosfomycin [11], nephrotoxicity for polymyxins [12] and resistance development for both [13,14].

The combined use of two antimicrobial agents has been used in the management of infectious diseases for decades, garnering more attention lately due to the aforementioned reasons. Combined treatment may prevent resistance selection, reduce dose-related toxicity as a result of reduced dosage of a specific compound, but more importantly in the case of MDR Gram-negatives, it is expected to provide a probable synergy between the two antimicrobials. On the other hand, potential disadvantages may include the increased cost, a greater risk for combined toxicity and the development of even more resistant bacteria [15]. Clinicians are increasingly prescribing combination therapy for the treatment of carbapenem-resistant Gram-negative bacteria according to a recent survey in large hospitals in Europe and the United States [16]. However, they are often driven empirically to the selection of the combined antimicrobials based on trial which may lead to inadequate patient care. A recent meta-analysis showed that synergy-guided antimicrobial combination therapy against MDR-GNB was significantly associated with survival [17].

Over the past years, *A. baumanni* and *K. pneumoniae* have emerged as serious nosocomial pathogens especially due to their extensively resistant antimicrobial profile [18]. Polymyxin (colistin or polymyxin B) is currently used as one of the last resort agents to treat the related infections, but resistance because of monotherapy urges the need to find effective antimicrobial combinations to overcome this problem. The combinations used most commonly include a polymyxin together with a carbapenem [16]. In the present study, we retrospectively evaluated the in vitro effectiveness of selected antimicrobial combinations against carbapenem- and colistin-resistant *A. baumannii* and *K. pneumoniae* clinical isolates.

## 2. Results

### 2.1. Acinetobacter baumannii

**Figure 1:**
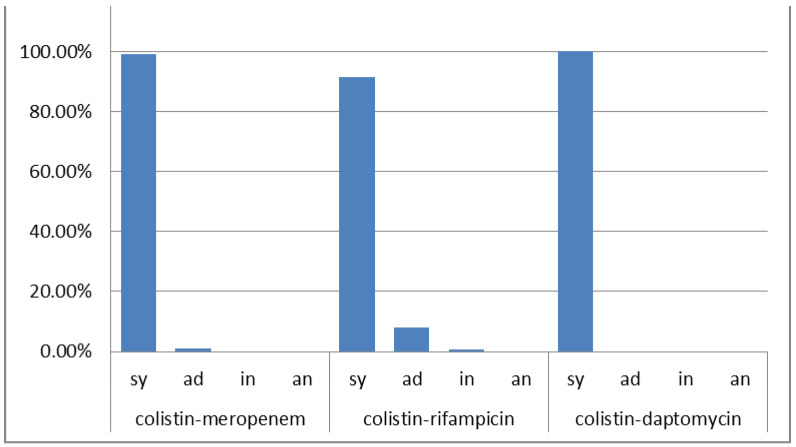
The% results of antimicrobial combinations tested for *A. baumannii*. sy: synergy; ad: additivity; in: indifference; an: antagonism.

The studied isolates displayed high rates of resistance to major classes of antimicrobials with 100% resistance to carbapenems and colistin (Table 1). The MIC_50_ and MIC_90_ for tigecycline were 3 mg/L and 6 mg/L; for ampicillin/sulbactam, ≥32 mg/L and ≥32 mg/L; for rifampicin, 6 mg/L and 32 mg/L; and for daptomycin, ≥256 mg/L and ≥256 mg/L, respectively. One hundred and ninety-eight isolates were tested for the colistin–meropenem combination exhibiting 98.99% (196/198) synergy (FICI range = 0.001–0.5) and 1.01% (2/198) additivity (FICI = 0.563). Although rifampicin and daptomycin are typically inactive against Gram-negative bacteria, high synergy rates were observed using the colistin–rifampicin combination with 91.52% (162/177) synergy (FICI range = 0.002–0.5); 7.91% (14/177) additivity (FICI range = 0.52–0.917) and 0.57% (1/177) indifference (FICI = 1.125). The colistin–daptomycin combination was tested in 129 isolates, resulting in 100% synergy (FICI range = 0.002–0.5) (Supplementary Table S1 and Figure 1).

### 2.2. Klebsiella pneumoniae

**Figure 2:**
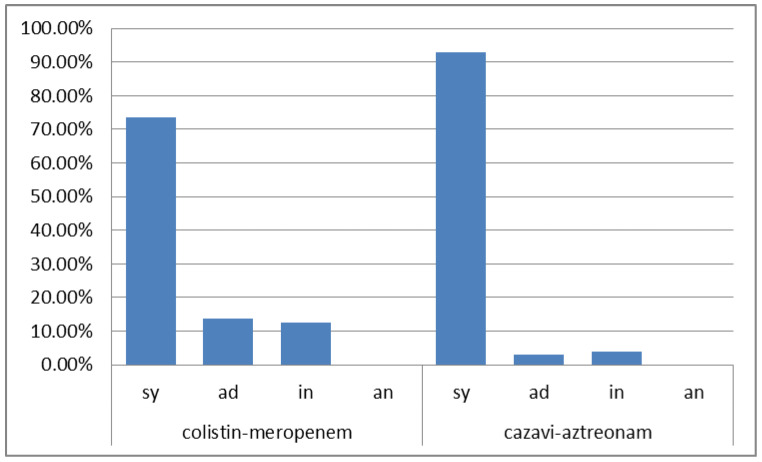
The % results of antimicrobial combinations tested for *K. pneumoniae*. cazavi: ceftazidime/avibactam; sy: synergy; ad: additivity; in: indifference; an: antagonism.

The *K. pneumoniae* isolates presented 100% resistance to carbapenems and colistin (Table 2). The resistance rate to ceftazidime/avibactam was 87.50% (the MIC_50_ and MIC_90_ were both ≥16 mg/L). Sixty-four were metallo-β-lactamase (MBL) producers, 13 were *Klebsiella pneumoniae* carbapenemase (KPC) producers and 29 were positive for both carbapenemase types. Eighty-seven isolates were tested for the colistin–meropenem combination exhibiting 73.56% (64/87) synergy (FICI range = 0.014–0.5); 13.80% (12/87) additivity (FICI range = 0.75–0.938); and 12.64% (11/87) indifference (FICI range = 1–2). Specifically, synergy rates of 66.7% (34/51), 90.9% (10/13) and 80% (20/25) were observed for MBL, KPC and MBL+KPC strains, respectively. For the ceftazidime/avibactam combination with aztreonam, the following were shown: 93% (93/100) synergy (FICI range = 0.0007–0.5); 3% (3/100) additivity (FICI range = 0.625–0.938); and 4% (4/100) indifference (FICI range = 1.25–4); no antagonism was observed (Supplementary Table S2 and Figure 2). Of the 62 MBL strains tested for the combination ceftazidime/avibactam with aztreonam, 95.2% (59/62) exhibited synergy, 3.2% (2/62) exhibited additivity and 1.6% (1/62) showed indifference, while all (10/10) of KPC-producing strains showed synergy. Lower rates of synergy, i.e., 85.7% (24/28), were observed for the strains with both carbapenemase types.

## 3. Discussion

According to the most recent epidemiological data from the Antimicrobial resistance Surveillance report in Europe, 21 countries, mostly in southern and eastern Europe, showed rates of *Acinetobacter* resistance to carbapenems equal to or above 50%, with 96.9% for Greece (https://www.ecdc.europa.eu/sites/default/files/documents/Surveillance-antimicrobial-resistance-in-Europe-2020.pdf) (accessed on 1 December 2022). This poses a great public health threat to patients and healthcare systems, with an estimated 2363 attributable deaths in 2015 in countries of the European Union (EU)/European Economic Area (EEA) [19]. Almost a quarter of EU/EEA countries reported carbapenem resistance percentages above 10% in *K. pneumonia*, while Greece had a rate of 73.7%.

Polymyxin, in some cases, is the only resort agent for the treatment of MDR and XDR Gram-negatives, but efficacy may be suboptimal in several infections according to the pharmacokinetic (PK) and pharmacodynamic (PD) data, even with the highest tolerable therapeutic dose [20]. Monotherapy may lead to resistance as well, probably due to the selection of pre-existing colistin-resistant subpopulations in heteroresistant strains [21] or emergence of chromosomal mutations besides the transmission of plasmid-mediated resistance [15,22,23,24]. Increased rates of colistin resistance have been reported all over the world, especially in Eastern Mediterranean countries and South East Asia, with a rate of 4% for Greece in the period2012–2016 [25]. Resistance to colistin was 47.7% among *A. baumannii* isolates from patients with ventilator-associated pneumonia in Greece, Italy and Spain [26]. According to a recent meta-analysis by Karakonstantis et al., the pooled rate of *A. baumannii* colistin heteroresistance was 33% [24]. Specifically for *K. pneumoniae* isolated from bloodstream infections, the pooled rate of resistance was increased to 12.90% for studies in 2020 and beyond, compared to 2.89% in the period 2015–2019 [27]. 

Combination regimens with colistin have been proposed to overcome the re-growth after colistin monotherapy either by reducing resistance or by enhancing bacterial killing through synergy between the two antimicrobials. Better antimicrobial effect is achieved by sub-population or mechanistic synergy that can act concomitantly. Sub-population synergy is a process where the resistant sub-populations of one antimicrobial are killed by the other and the opposite. Mechanistic synergy refers to two antimicrobials with different mode of action that enhance the killing of one another. Colistin, for example, seems to increase the permeability of the outer membrane of Gram-negatives.

It should be pointed out that methods for synergy testing are not completely standardized, and there are variations concerning the interpretation of synergy [28]. Most studies use time-kill assays and checkerboard as these are considered standard methods for antimicrobial combinations testing [28,29]. These are, however, time-consuming and laborious for a clinical microbiology laboratory. Gradient diffusion methods are widely used and easy to perform and, thus, can be more easily integrated in a routine base for synergy testing [28]. Since our laboratory is a clinical diagnostic lab, we chose the MIC gradient synergy testing due to the increased daily workload. For synergy interpretation, we used the most recent criteria of antagonism defined as FICI > 4 [15].

Antimicrobials selected for synergy in our study were bactericidal, since a recent meta-analysis showed that combinations including bactericidal antimicrobials had better synergy rates, while most antagonistic effects were demonstrated when a bacteriostatic antimicrobial was included [28]. 

Recently published studies demonstrated in vitro synergistic effect for the combinations of polymyxin with a carbapenem, rifampicin or a glycopeptide for colistin-susceptible but also colistin-resistant MDR or XDR *A. baumannii* isolates [30]. On the contrary, multiple studies testing colistin paired with tigecycline failed to achieve synergy in vitro and in vivo compared with polymyxin/carbapenem combinations [31] and resulted in a lesser microbiological cure [32]. A systematic review and meta-analysis that included only killing assay (PK/PD and time-killing) studies showed high level of synergy for polymyxin/meropenem and polymyxin-rifampicin combinations against *A. baumannii* isolates [33].

The combinations used most include a polymyxin together with a carbapenem. Systematic reviews and meta-analysis with *A. baumannii* strains showed pooled synergy rates of 17.5–98.3% for polymyxin-carbapenem combinations [34,35,36]. The great fluctuation is depending on the different applied method for synergy, with higher rates reported for time-kill assays but also on the number of isolates tested, their different susceptibility profile and the clonal diversity of strains [36,37]. The synergy rate for meropenem was higher than that of imipenem (85.2–86% vs. 56–66.2%, respectively). For polymyxin-resistant strains, the synergy rate was above 50% [34,36]. Our study exhibited a high rate 98.99% of synergy for *A. baumannii* strains against the combination of colistin–meropenem, similarly to the 96% rate of a recent study with colistin-resistant strains [38]. A study that compared colistin–meropenem against colistin-resistant (CoR) and colistin-susceptible (CoS) *A. baumannii* isolates showed increased rates of synergy for the CoR group (85.4% vs. 4.9% for the CoS group) [39]. Low rates of antagonism were observed in previous studies [36], whereas none of our strains exhibited antagonism. 

Recent studies pointed out the paradoxical phenomenon of CoR Gram-negatives strains showing increased susceptibility to drugs usually inactive against Gram-negatives such as rifampicin, daptomycin, glycopeptides or macrolides [15]. A possible explanation might be the increased permeability due to the alteration of the outer membrane which allows the entrance of those drugs. Data from systematic reviews and meta-analyses showed high rates of synergy for the pair polymyxin-rifampicin [30,33,34] and specifically for CoR strains 56.8%, similarlyto CoS. Three randomized controlled trials showed that colistin–rifampicin managed an increased rate of microbiological eradication but had no effect on mortality or length of hospitalization [40,41,42]. A study with CoR *A. baumannii* strains exhibited higher synergy rates than CoS for the colistin–rifampicin pair (80.5% vs. 14.6% respectively) [39]. This is in accordance with the high rate of synergy 91.52% observed in our CoR strains. Decreased values of MICs of rifampicin alone were observed in our study (MIC_5_), similarly to one study with CoR strains [43]. 

Colistin combined with daptomycin has proved very efficient against our CoR *A. baumannii* strains with 100% synergy. On the contrary, a study evaluating this combination against XDR *Acinetobacter* strains with time-kill assays showed synergistic effect only against CoS and indifference against CoR, but the different synergy methodology must be taken into account [44]. Few studies have evaluated this combination; however, no antagonism was observed [44,45,46]. To the best of our knowledge, our collection is the largest evaluating the colistin–daptomycin combination.

The most prevalent mechanism of resistance for *K. pneumoniae* is the production of β-lactamases with a geographical distribution [47]. The novel β-lactam/β-lactamase inhibitor combinations are used against non-metallo-β-lactamase-producing strains, but for MBL-producers, the treatment choices are limited. Many studies have proposed the combination of ceftazidime/avibactam plus aztreonam for MBL strains with high synergy rates [48,49,50,51,52,53]. As aztreonam is not hydrolyzed by MBLs, the addition of avibactam can inhibit other β-lactamases (ESBLs, AmpCs, serine carbapenemases) if present and thus restore the susceptibility to aztreonam [49,54]. In our study, 87.65% showed synergy to this combination in accordance with a study including only CoR carbapenem-resistant isolates [55]. Specifically for MBL-producing strains a rate of 95.2% was observed, while strains with both carbapenemase types had a lower rate of 85.7%. As expected, the combination exhibited synergistic effect for the small number of KPC isolates tested as they are already susceptible to ceftazidime/avibactam. An observational prospective study in patients with bloodstream infections caused by MBL-producing Enterobacterales, mainly *K. pneumoniae*, showed better clinical response for the ceftazidime/avibactam plus aztreonam combination than other therapeutic agents [56]. The Infectious Diseases Society of America (IDSA) recommends this combination for the treatment of MBL-producing CRE (https://www.idsociety.org/practice-guideline/amr-guidance/) (accessed on 1 December 2022), while the aztreonam/avibactam drug combination is pending a phase III clinical trial (https://www.clinicaltrialsregister.eu/ctr-search/search?query=aztreonam-avibactam) (accessed on 1 December 2022). Meanwhile, many studies have proved the efficacy of aztreonam/avibactam for the treatment of CRE, including MBLproduction [57,58,59].

The pooled synergy rate for the combination of colistin–carbapenem against *K. pneumoniae* was 44% in a meta-analysis, and when examining CoR *K. pneumoniae* isolates, the rate increased to 62% [36]. A synergy rate of 73.56% for the combination of colistin plus meropenem was observed in our study. Although KPC is the predominant mechanism of resistance for *K. pneumoniae* strains in our hospital (data not shown) we only included 13 KPC strains, as ceftazidime/avibactam can be used as a therapy for these isolates. This drug, however, may not be available in every hospital; thus, alternative therapeutic options must be taken into account. With a synergy rate of 90.9% in our study, colistin–meropenem could be used in the absence of ceftazidime/avibactam. Lower rates were observed for MBL (66.7%, 34/51) and MBL+KPC strains (80%, 20/25), indicating that the combination of ceftazidime/avibactam plus aztreonam is more synergistic than colistin–meropenem.

Randomized controlled trials failed to show reduction in all-over mortality in the group of patients receiving combination therapy compared to colistin monotherapy [40,41,42,60]. A multinational observational retrospective study among patients with CRE bloodstream infections (INCREMENT) demonstrated that combination therapy was associated with lower mortality than monotherapy only in patients with a high mortality score [61]. On the other hand, colistin combinations with carbapenems, rifampicin and sulbactam were related to a higher microbiological effect compared to colistin monotherapy against *A. baumannii* strains [32,62]. This may be due to the fact that microbiological response represents the effect of the drug, but other factors might be responsible for the clinical deterioration [32]. Interestingly, a lower mortality rate was observed in the subgroup of CoR *Acinetobacter* strains of the AIDA study for colistin–meropenem combination compared to colistin monotherapy [63]. Resistance to colistin usually contributes to fitness cost, and the administration of meropenem may restore virulence through gene expression changes. Unfortunately, data on synergy were not reported on this subgroup [63]. This would be significant, as the results of combination synergy against particular isolates does not reflect all *Acinetobacter* strains [64]. Further studies are needed to support this result. Discrepancy between in vitro testing and clinical trial results may be due to the pharmacokinetics of colistin with a great variability especially among critically ill patients, the concomitant co-morbidities, the specific pathogen and resistance mechanism, the site of infection (the respiratory tract is not easily accessible either for colistin or other antimicrobials) and the delay on the administration of empirical treatment [15,65].

Our study presents some limitations. First of all, our results refer to the in vitro activity of the studied antimicrobial combinations and should be interpreted in the context that in vitro susceptibility data are not the only parameter that has to be taken into account when deciding the proper antimicrobial treatment for each patient. Clinical management is a dynamic process with individualized adjustment chemotherapy over time. Second, even though we included only single-patient isolates, we were not able to employ sequencing-based methods to better characterize the molecular epidemiology of the strains implemented in our study. Third, it is well known that diffusion methods are generally not recommended for colistin, because its large molecule does not diffuse as much as other antimicrobials in agar plates. However, the MIC test strip fixed-ratio method is acceptably labor intensive for clinical laboratories and is used for the in vitro synergistic activity testing of antimicrobial combinations including colistin [39]. Finally, our work is a single-center study and does not necessarily reflect the whole picture regarding the susceptibility of strains isolated in other institutions. Therefore, we strongly recommend the antimicrobial combination testing for each XDR or PDR isolate, especially in cases presenting resistance to polymyxins.

## 4. Materials and Methods

### 4.1. Study Design

Two hundred *A. baumannii* single clinical isolates with resistance to carbapenems and colistin between 1 January 2021 and 31 July 2022 were included in the study; 81 were isolated from blood, 76 from bronchoalveolar secretions, 21 from urine, 7 from sputum, 6 from central lines, 4 from wound cultures, 3 from biopsy and soft tissues, and 1 from pus, pleural fluid and cerebrospinal fluid, respectively. A total of 198 isolates were tested for colistin and meropenem synergy; 177 were tested for the colistin and rifampicin combination; and 129 were tested for colistin and daptomycin.

A total of 106 *K. pneumoniae* single clinical isolates with resistance to carbapenems and colistin were also included; 32 were isolated from bronchoalveolar secretions, 31 from urine, 30 from blood, 8 from central line catheters, 4 from wound infections and 1 from sputum. Overall, 87 were tested for colistin and meropenem synergy and 100 for the ceftazidime/avibactam plus aztreonam combination.

Antimicrobial susceptibility testing was performed by the Vitek2 (bioMérieux, Marcy-l’Étoile, France), where applicable. Tigecycline, rifampicin and daptomycin were tested with MIC test strips (Liofilchem, Roseto degli Abruzzi, Teramo, Italy). Colistin susceptibility was performed by the broth microdilution method (Liofilchem, Roseto degli Abruzzi, Teramo, Italy). MIC ranges, MIC_50_ and MIC_90_ were calculated for the antimicrobials tested. Antimicrobial resistance rates were calculated according to the EUCAST breakpoints v 12.0 (2022). In vitro synergistic activity testing of antimicrobial combinations was performed using the MIC test strip fixed-ratio method.

### 4.2. MIC Test Strip Fixed-Ratio Method

The MIC test strip fixed-ratio method [37] was used for the synergistic activity of antimicrobial combinations using MIC test strips of both antimicrobials for each antimicrobial combination. Three antimicrobial combinations of colistin with either meropenem or rifampicin or daptomycin were tested for *A. baumannii*. Colistin with meropenem and ceftazidime/avibactam with aztreonam were tested for *K. pneumoniae*. Briefly, a 0.5 McFarland solution was prepared and inoculated onto a Mueller Hinton agar plate. The MIC strip of the first antimicrobial (antimicrobial agent A) was placed and left for 1 h, at room temperature, to allow the antimicrobial to diffuse into the medium. Afterwards, the MIC strip of antimicrobial A was removed, cleaned with alcohol and saved as MIC template reading scale. The MIC strip of the second antimicrobial (antimicrobial agent B) was then placed directly over the imprint of A with the highest concentrations coinciding. In parallel, plates with an MIC strip of each antimicrobial alone were prepared. The plates were incubated, at 36–37 °C, for 18–24 h, and the MICs of each drug alone along with the MIC of the drugs in combination were assessed with the use of the respective MIC strip/scales. The results were interpreted using the fractional inhibitory concentration index (FICI) [66] calculated as:

FICI = FIC_agentA_ + FIC_agentB_ = MIC_AB_/MIC_A_ + MIC_BA_/MIC_B_ (1)

MIC_AB_ is the MIC of A in the presence of B; MIC_BA_ is the MIC of B in the presence of A; MIC_A_ and MIC_B_ are the MICs of each drug alone. ‘Synergy’, ‘additivity’, ‘indifference’ and ‘antagonism’ were interpreted when the FICI was ≤0.5, >0.5–≤1, >1–≤4 and >4, respectively. Synergy is considered the interaction of the two antimicrobials to increase each other’s effect; additivity means the additional effect of the action of two antimicrobials without synergism; antagonism suggests that the combined effect of the two antimicrobials is less than the most effective one used individually; and indifference indicates the absence of all the aforementioned phenomena.

### 4.3. Phenotypic Detection of K. pneumoniae Carbapenem Resistance Mechanisms

For the phenotypic detection of MBL or KPC production, the double meropenem disc test was used. The double meropenem disc test is a combined disc test using meropenem discs with and without the carbapenemase inhibitors EDTA and phenylboronic acid. Briefly, a 0.5 McFarland bacterial suspension was prepared and inoculated onto a Mueller Hinton agar plate. Four meropenem discs were placed on the surface of the agar. One was left without inhibitors. On the second disc, 10 μL of EDTA 0.1 M was added. Phenylboronic acid (20 g/L) was added on the third disc. Finally, both inhibitors were added on the fourth disc. After 18–24 h of incubation, the evaluation of the result was performed as follows: The absence of inhibition zone around the first disc or an inhibition zone of <22 mm indicated carbapenem resistance. The presence of an inhibition zone around the second and the fourth disc with a diameter ≥5 mm wider than that of the first disc was indicative of MBL production. The presence of an inhibition zone around the third and the fourth disc with a diameter ≥5 mm wider than that of the first disc was indicative of KPC production. The presence of an inhibition zone around the second and third disc with a diameter ≥5 mm wider than that of the first disc and an even larger inhibition zone around the fourth disc was indicative of both MBL and KPC production.

## 5. Conclusions

In vitro colistin-based combinations with either meropenem or rifampicin or daptomycin resulted in high synergy rates, rendering them a valuable option for the treatment of colistin-resistant *A. baumannii* infections. The same applies for ceftazidime/avibactam-aztreonam and colistin–meropenem combinations against difficult to treat *K. pneumoniae* infections. MIC gradient synergy testing can serve as a simple tool in clinical microbiological laboratories guiding clinicians to the proper therapy for these resistant pathogens.

## Figures and Tables

**Table 1:** Antimicrobial profile of *A. baumannii* isolates. NA: not applicable.

Antimicrobial	Number of Isolates Tested	MIC Range (mg/L)	MIC_50_ (mg/L)	MIC_90_ (mg/L)	Resistance (%)
Meropenem	200	8–≥16	≥16	≥16	100
Imipenem	200	≥16	≥16	≥16	100
Colistin	200	4–≥16	≥16	≥16	100
Ciprofloxacin	200	≥4	≥4	≥4	100
Amikacin	136	4–≥64	≥64	≥64	97.79
Gentamicin	133	1–≥16	≥16	≥16	98.49
Ampicillin/Sulbactam	158	16–≥32	≥32	≥32	NA
Tigecycline	192	0.047–12	3	6	NA
Rifampicin	178	1–≥256	6	32	NA
Daptomycin	128	≥256	≥256	≥256	NA

**Table 2:** Antimicrobial profile of *K. pneumoniae* isolates. NA: not applicable.

Antimicrobial	Number of Isolates Tested	MIC Range (mg/L)	MIC_50_ (mg/L)	MIC_90_ (mg/L)	Resistance (%)
Meropenem	106	≥16	≥16	≥16	100
Imipenem	106	≥16	≥16	≥16	100
Colistin	106	4–≥16	≥16	≥16	100
Ceftazidime/Avibactam	104	1–≥16	≥16	≥16	87.50
Ceftazidime	103	16–≥64	≥64	≥64	100
Ceftolozane/Tazobactam	83	≥32	≥32	≥32	100
Cefotaxime	81	2–≥64	≥64	≥64	96.29
Aztreonam	104	16–≥64	≥64	≥64	100
Ciprofloxacin	102	0.25–≥4	≥4	≥4	99.01
Amikacin	104	2–≥64	32	≥64	97.11
Gentamicin	101	1–≥16	≥16	≥16	93.06
Piperacillin/Tazobactam	102	≥128	≥128	≥128	100
Fosfomycin	102	16–≥256	256	≥256	90.19
Tigecycline	92	0.25–8	2	8	NA
Chloramphenicol	91	2–≥64	32	≥64	87.91

## Data Availability

Not applicable.

## Supplementary Materials

The following supporting information can be downloaded at: https://www.mdpi.com/article/10.3390/antibiotics12010093/s1, Table S1: Results of antimicrobial combinations tested for *A. baumannii*; Table S2: Results of antimicrobial combinations tested for *K. pneumoniae*.
