# Supplementary material for: In Vitro Synergistic Activity of Antimicrobial Combinations against Carbapenem- and Colistin-Resistant Acinetobacter baumannii and Klebsiella pneumoniae
Source: Antibiotics (Basel). 2023 Jan 5;12(1):93. doi: 10.3390/antibiotics12010093 (PMC9855173; doi:10.3390/antibiotics12010093)
Supplement: Supplementary file 1 [file antibiotics-12-00093-s001.zip › antibiotics-2131577-supplementary.pdf]

Supplementary material

Supplementary Table S1. Results of antimicrobial combinations tested for *A. baumannii*. FICI: fractional inhibitory concentration index; sy: synergy; ad: additivity; in: indifference; an: antagonism.

A. baumannii isolate accession number	Antimicrobial combination					
	colistin + meropenem FICI (interpretation)		colistin + rifampicin FICI (interpretation)		colistin + daptomycin FICI (interpretation)	
67538	0.282	sy	0.182	sy		
227561	0.469	sy	0.465	sy	0.260	sy
76511	0.141	sy	0.087	sy		
90009	0.030	sy	0.130	sy	0.190	sy
33391	0.201	sy				
117554	0.040	sy	0.070	sy	0.030	sy
3793	0.313	sy	0.135	sy	0.258	sy
131000	0.050	sy	0.100	sy	0.020	sy
100847	0.313	sy	0.066	sy		
120567	0.375	sy	0.125	sy		
197616	0.047	sy	0.006	sy	0.066	sy
232959	0.281	sy	0.563	ad	0.266	sy
65780	0.140	sy	0.158	sy	0.190	sy
118411	0.080	sy	0.070	sy	0.050	sy
3626	0.468	sy	0.280	sy	0.387	sy
229383	0.500	sy	0.313	sy	0.310	sy
31463	0.022	sy	0.172	sy	0.200	sy
189928	0.008	sy	0.078	sy		
123900	0.500	sy	0.127	sy		
143702	0.250	sy				
263605	0.310	sy	0.440	sy	0.385	sy
236413	0.313	sy	0.625	ad	0.500	sy
79534	0.016	sy	0.104	sy	0.105	sy
267318	0.469	sy	0.500	sy	0.198	sy
130692	0.469	sy				
95924	0.010	sy	0.100	sy	0.050	sy
95890	0.107	sy	0.104	sy		
142010	0.469	sy				
270332	0.325	sy	0.313	sy	0.250	sy
111940	0.100	sy	0.050	sy	0.030	sy
263832	0.470	sy	0.300	sy	0.500	sy
96495	0.125	sy	0.049	sy		
171482	0.094	sy				
144145	0.375	sy				
31100	0.020	sy	0.420	sy	0.140	sy
84693	0.094	sy	0.141	sy	0.398	sy
105788	0.040	sy	0.100	sy	0.020	sy
151812	0.313	sy				
260699	0.500	sy	0.844	ad	0.200	sy
90065	0.059	sy	0.087	sy		
203083	0.015	sy	0.007	sy	0.097	sy
153247	0.313	sy				
238043	0.060	sy	0.070	sy	0.100	sy
131040	0.050	sy	0.050	sy	0.030	sy
58521	0.250	sy	0.421	sy	0.140	sy
104710	0.040	sy	0.050	sy	0.030	sy
272393	0.500	sy	0.422	sy	0.280	sy

52315	0.107	sy				
270328	0.375	sy	0.437	sy	0.385	sy
263393	0.190	sy	0.520	ad	0.140	sy
223177	0.469	sy	0.107	sy		
113303	0.188	sy	0.049	sy		
148812	0.050	sy	0.500	sy	0.030	sy
139070	0.250	sy				
229283	0.188	sy	0.469	sy	0.266	sy
133520	0.500	sy	0.100	sy	0.050	sy
70751	0.116	sy	0.197	sy	0.002	sy
268862	0.094	sy	0.438	sy	0.258	sy
263547	0.188	sy	0.395	sy	0.199	sy
43676	0.023	sy	0.400	sy	0.200	sy
190505	0.008	sy	0.002	sy	0.035	sy
32038	0.046	sy	0.256	sy	0.140	sy
125054	0.500	sy	0.145	sy		
232904	0.188	sy	0.396	sy	0.105	sy
86086	0.281	sy	0.094	sy		
224302	0.188	sy	0.049	sy		
95895	0.047	sy	0.057	sy		
92139	0.035	sy	0.395	sy	0.199	sy
204909	0.024	sy	0.005	sy		
197610	0.016	sy	0.010	sy	0.105	sy
197809	0.094	sy	0.002	sy	0.422	sy
270325	0.313	sy	0.375	sy	0.385	sy
31983	0.058	sy	0.240	sy	0.250	sy
60026	0.125	sy	0.280	sy	0.051	sy
30768	0.034	sy	0.469	sy	0.190	sy
110420	0.030	sy	0.200	sy	0.030	sy
125740	0.125	sy				
185612	0.016	sy	0.020	sy	0.035	sy
65534	0.250	sy	0.420	sy	0.070	sy
275944	0.469	sy	0.198	sy	0.198	sy
176461	0.039	sy				
277734	0.563	ad	0.795	ad	0.265	sy
201158	0.031	sy	0.064	sy	0.018	sy
51352	0.313	sy	0.375	sy	0.258	sy
89286	0.078	sy	0.111	sy		
72340	0.140	sy	0.188	sy	0.190	sy
191991	0.001	sy	0.020	sy	0.035	sy
275939	0.375	sy	0.123	sy	0.203	sy
197624	0.003	sy	0.026	sy	0.035	sy
75867	0.375	sy	0.343	sy	0.200	sy
278773	0.468	sy	0.257	sy	0.257	sy
275380	0.250	sy	0.563	ad	0.105	sy
180180	0.080	sy	0.050	sy	0.050	sy
196677	0.035	sy	0.037	sy	0.127	sy
197874	0.016	sy	0.022	sy	0.053	sy
179117	0.031	sy	0.127	sy		
1009533	0.469	sy	0.470	sy	0.265	sy
2381	0.187	sy	0.375	sy	0.199	sy
277736	0.563	ad	0.465	sy	0.386	sy
86077	0.070	sy	0.104	sy		
125361	0.020	sy	0.050	sy	0.020	sy
187704	0.094	sy	0.003	sy	0.035	sy
272364	0.220	sy	0.375	sy	0.375	sy
226213	0.375	sy	0.281	sy	0.266	sy
134571	0.020	sy	0.100	sy	0.020	sy

89101	0.070	sy	0.071	sy		
189794	0.031	sy	0.018	sy	0.035	sy
236499	0.313	sy				
30192	0.281	sy				
259994	0.281	sy	0.469	sy	0.260	sy
121035	0.040	sy	0.300	sy	0.030	sy
277735	0.313	sy	0.437	sy	0.258	sy
112461	0.313	sy	0.071	sy		
279644	0.375	sy	0.688	ad	0.398	sy
279922	0.469	sy	0.469	sy	0.386	sy
29283	0.110	sy	0.465	sy	0.500	sy
270284	0.500	sy	0.310	sy	0.203	sy
127132	0.020	sy	0.020	sy	0.020	sy
198386	0.020	sy	0.012	sy	0.129	sy
113448	0.313	sy	0.094	sy		
102488	0.188	sy	0.049	sy		
192722	0.003	sy	0.005	sy	0.026	sy
90601	0.141	sy	0.104	sy		
97373	0.125	sy	0.186	sy		
114294	0.125	sy	0.049	sy		
1015	0.375	sy	0.917	ad	0.398	sy
270330	0.469	sy	0.469	sy	0.327	sy
181282	0.015	sy	0.012	sy		
117201	0.281	sy	0.039	sy		
90620	0.107	sy	0.138	sy		
1010672	0.375	sy	0.844	ad	0.267	sy
126245	0.500	sy				
44922	0.046	sy	0.281	sy	0.210	sy
97054	0.469	sy	0.073	sy		
27419	0.036	sy	0.460	sy	0.200	sy
93496	0.107	sy	0.125	sy		
124031	0.375	sy				
72358	0.148	sy	0.084	sy		
6311	0.258	sy	0.258	sy	0.258	sy
197942	0.039	sy	0.009	sy	0.258	sy
27416	0.100	sy	0.469	sy	0.265	sy
44532	0.281	sy	0.159	sy		
192492	0.012	sy	0.050	sy	0.070	sy
99296	0.125	sy	0.049	sy		
225334	0.281	sy	0.180	sy		
44863	0.201	sy	0.111	sy		
31977	0.300	sy	0.465	sy	0.265	sy
79413	0.281	sy	0.104	sy		
31632	0.110	sy	0.465	sy	0.265	sy
36685	0.140	sy	0.437	sy	0.265	sy
190352	0.031	sy	0.005	sy	0.070	sy
176209	0.031	sy				
48759			0.460	sy	0.258	sy
257357	0.375	sy			0.422	sy
258542	0.375	sy			0.422	sy
288824	0.281	sy	0.437	sy	0.387	sy
82510	0.023	sy	0.094	sy	0.133	sy
131253	0.060	sy	0.060	sy	0.100	sy
503673	0.281	sy				
7758	0.387	sy	0.795	ad	0.387	sy
70418	0.078	sy	0.150	sy	0.064	sy
206970	0.063	sy	0.017	sy		
49258			0.250	sy	0.265	sy

33727	0.140	sy	0.465	sy	0.500	sy
194762	0.004	sy	0.005	sy	0.060	sy
28965	0.180	sy	0.593	ad	0.280	sy
91232	0.039	sy	0.039	sy		
204577	0.094	sy	0.120	sy		
204577	0.094	sy	0.120	sy		
68381	0.018	sy	0.098	sy	0.099	sy
80228	0.049	sy	0.219	sy	0.398	sy
80198	0.031	sy	0.094	sy	0.141	sy
229534	0.188	sy	0.469	sy	0.199	sy
100343	0.375	sy	0.057	sy		
113254	0.500	sy	0.075	sy		
204600	0.018	sy	0.047	sy		
69021	0.148	sy	0.020	sy		
169441	0.125	sy				
81018	0.053	sy	0.470	sy		
71313	0.023	sy	0.026	sy	0.025	sy
33732	0.039	sy	0.688	ad	0.257	sy
58059	0.070	sy	0.750	ad	0.508	sy
91183	0.047	sy	0.059	sy		
270320	0.180	sy	0.310	sy	0.250	sy
64048	0.188	sy	1.125	in	0.260	sy
148141	0.050	sy	0.015	sy	0.030	sy
199874	0.031	sy	0.030	sy	0.105	sy
164745	0.375	sy				
182722	0.012	sy	0.009	sy		
181431	0.003	sy				
190759	0.008	sy	0.019	sy	0.018	sy
209208	0.059	sy	0.086	sy		
62569	0.188	sy	0.435	sy	0.012	sy
63987	0.375	sy	0.688	ad	0.387	sy
48026	0.420	sy	0.469	sy	0.193	sy
199586	0.013	sy	0.009	sy	0.063	sy
89330	0.047	sy	0.099	sy		
72532	0.469	sy	0.146	sy	0.190	sy
109531	0.030	sy	0.100	sy	0.030	sy
96720	0.004	sy	0.013	sy	0.030	sy

Supplementary Table S2. Results of antimicrobial combinations tested for *K. pneumoniae*. FICI: fractional inhibitory concentration index; cazavi: ceftazidime/avibactam; sy: synergy; ad: additivity; in: indifference; an: antagonism; MBL: metallo-β-lactamase; KPC: *Klebsiella pneumoniae* carbapenemase.

K. pneumoniae isolate accession number	Phenotypically detected resistance mechanism	Antimicrobial combination			
		colistin + meropenem FICI (interpretation)		cazavi + aztreonam FICI (interpretation)	
5443	MBL	0.313	sy		
55261	MBL	0.233	sy		
67868	MBL	0.92	ad	0.003	sy
75416	MBL, KPC	0.172	sy	0.0007	sy
91223	MBL			0.001	sy
93696	MBL			0.023	sy
98970	MBL, KPC	0.5	sy	0.01	sy
101784	MBL			0.006	sy
103075	MBL	0.75	ad	0.074	sy
102603	MBL	0.5	sy	0.003	sy
105833	MBL	0.5	sy	0.009	sy
106997	MBL	0.5	sy	0.003	sy
108107	MBL, KPC			0.003	sy
107528	MBL, KPC			0.006	sy
113425	MBL, KPC	0.375	sy	0.003	sy
118810	MBL	0.375	sy	0.4	sy
118117	MBL	0.5	sy	0.04	sy
120360	MBL, KPC	0.5	sy	0.004	sy
122855	MBL, KPC	0.75	ad	0.03	sy
130082	MBL	0.75	ad	0.0008	sy
129338	MBL	0.5	sy	0.001	sy
132171	MBL	0.375	sy	0.009	sy
140735	MBL	0.375	sy	0.004	sy
151864	MBL, KPC	0.375	sy	0.003	sy
151628	MBL, KPC	0.375	sy	0.007	sy
155646	MBL	0.75	ad	0.048	sy
160399	MBL	0.75	ad	0.097	sy
160782	MBL, KPC	0.25	sy	1.56	in
161199	MBL, KPC	0.5	sy	0.005	sy
162193	MBL, KPC	0.375	sy	0.038	sy
162830	KPC	0.313	sy	0.032	sy
166265	MBL, KPC	0.5	sy		
166123	MBL, KPC	0.5	sy	0.01	sy
168860	KPC	0.022	sy		
179049	MBL	0.375	sy	0.220	sy
181425	KPC	0.047	sy		
181671	KPC	0.039	sy		
181630	MBL, KPC	0.047	sy	0.004	sy
184336	MBL, KPC	0.100	sy	1.250	in
186706	MBL, KPC	0.092	sy	0.029	sy
186399	MBL, KPC	0.092	sy	0.018	sy
187704	MBL, KPC	0.092	sy	0.029	sy
194419	KPC	0.094	sy	0.06	sy
194761	MBL, KPC	0.014	sy	0.014	sy
198183	KPC	0.312	sy	0.126	sy
200308	KPC	0.250	sy	0.500	sy
203868	MBL	0.063	sy	0.001	sy
207584	MBL	0.375	sy	0.005	sy
207651	MBL	0.250	sy	0.015	sy

210453	MBL, KPC			0.034	sy
211308	MBL	0.375	sy	0.004	sy
211364	MBL	0.360	sy	0.007	sy
211319	KPC			0.098	sy
211123	MBL	0.281	sy	0.010	sy
212409	MBL	0.469	sy	0.039	sy
214234	MBL	0.188	sy	0.004	sy
217930	MBL			0.013	sy
217801	MBL			0.005	sy
218018	MBL, KPC			0.938	ad
218132	MBL			0.007	sy
229673	MBL	2.000	in	0.002	sy
236287	MBL			0.005	sy
243606	MBL			0.015	sy
259530	MBL	0.313	sy	0.022	sy
263614	MBL, KPC	1.000	in	0.009	sy
263381	MBL, KPC	0.750	ad	0.009	sy
263301	MBL	1.000	in	0.156	sy
270490	MBL	0.500	sy	0.310	sy
274570	MBL	0.750	ad	0.005	sy
276255	MBL	0.750	ad	0.033	sy
279894	MBL	1.500	in	0.029	sy
289041	MBL	1.000	in	0.038	sy
2168	MBL	0.375	sy	0.005	sy
6249	MBL	0.750	ad	0.029	sy
5799	MBL	1.000	in	0.003	sy
6265	MBL	1.000	in	0.003	sy
7766	MBL	0.500	sy	0.005	sy
12724	MBL			0.938	ad
18504	MBL			0.003	sy
19178	KPC			0.126	sy
21250	MBL			0.005	sy
21435	MBL			0.005	sy
33720	MBL, KPC	0.120	sy	0.005	sy
33747	MBL	0.078	sy	0.625	ad
39455	MBL	0.421	sy	0.038	sy
48734	MBL, KPC	0.094	sy	0.005	sy
48687	KPC	0.094	sy	0.126	sy
51340	KPC	1.125	in	0.039	sy
53603	MBL			0.005	sy
57305	MBL	0.250	sy	0.014	sy
58608	MBL	1.570	in	0.010	sy
61996	MBL, KPC	1.000	in	0.004	sy
66196	MBL	0.750	ad	0.005	sy
69763	MBL, KPC	1.000	in	0.004	sy
74042	MBL, KPC	0.313	sy	1.875	in
75938	MBL	0.938	ad	0.003	sy
82425	MBL	0.139	sy	0.015	sy
84690	MBL	0.188	sy	0.015	sy
88862	KPC	0.375	sy	0.383	sy
89008	MBL	0.069	sy	4.000	in
92029	KPC	0.031	sy	0.024	sy
94340	MBL	0.046	sy	0.005	sy
94561	MBL	0.025	sy	0.051	sy
95924	MBL	0.024	sy	0.030	sy
119233	MBL	0.063	sy	0.094	sy
145639	MBL	0.070	sy	0.020	sy
